# Spike desensitisation as a mechanism for high-contrast selectivity in retinal ganglion cells

**DOI:** 10.3389/fncel.2023.1337768

**Published:** 2024-01-10

**Authors:** Le Chang, Yanli Ran, Mingpo Yang, Olivia Auferkorte, Elisabeth Butz, Laura Hüser, Silke Haverkamp, Thomas Euler, Timm Schubert

**Affiliations:** ^1^Institute for Ophthalmic Research, University of Tübingen, Tübingen, Germany; ^2^Werner Reichardt Centre for Integrative Neuroscience (CIN), University of Tübingen, Tübingen, Germany; ^3^Key Laboratory of Primate Neurobiology, Institute of Neuroscience, CAS Center for Excellence in Brain Science and Intelligence Technology, Chinese Academy of Sciences, Shanghai, China; ^4^Key Laboratory of Preclinical Study for New Drugs of Gansu Province, and Institute of Physiology, School of Basic Medical Sciences, Lanzhou University, Lanzhou, China; ^5^Max-Planck-Institute for Brain Research, Frankfurt am Main, Germany; ^6^Department of Computational Neuroethology, Max Planck Institute for Neurobiology of Behavior – Caesar, Bonn, Germany

**Keywords:** retina, action potential, sodium channel, light response, retinal ganglion cell, spike generator, mouse

## Abstract

In the vertebrate retina, several dozens of parallel channels relay information about the visual world to the brain. These channels are represented by the different types of retinal ganglion cells (RGCs), whose responses are rendered selective for distinct sets of visual features by various mechanisms. These mechanisms can be roughly grouped into synaptic interactions and cell-intrinsic mechanisms, with the latter including dendritic morphology as well as ion channel complement and distribution. Here, we investigate how strongly ion channel complement can shape RGC output by comparing two mouse RGC types, the well-described ON alpha cell and a little-studied ON cell that is EGFP-labelled in the *Igfbp5* mouse line and displays an unusual selectivity for stimuli with high contrast. Using patch-clamp recordings and computational modelling, we show that a higher activation threshold and a pronounced slow inactivation of the voltage-gated Na^+^ channels contribute to the distinct contrast tuning and transient responses in ON *Igfbp5* RGCs, respectively. In contrast, such a mechanism could not be observed in ON alpha cells. This study provides an example for the powerful role that the last stage of retinal processing can play in shaping RGC responses.

## 1 Introduction

Retinal ganglion cells (RGCs) and their upstream circuits detect and encode specific visual features and relay this information along parallel pathways to higher visual centres in the brain [reviewed in (Kerschensteiner, 2022)]. Functional, anatomical, and genetic evidence (Baden et al., 2016; Bae et al., 2018; Rheaume et al., 2018; Goetz et al., 2021) support the presence of at least 40 RGC types in the mouse retina.

Retinal ganglion cells receive their excitatory drive mostly from bipolar cells (BCs), which relay the photoreceptor signal to the inner retina, and their inhibitory input from amacrine cells (ACs) [reviewed in (Diamond, 2017)]. As the dendrites of different RGC types arborize at distinct inner plexiform layer (IPL) depths (Helmstaedter et al., 2013; Sümbül et al., 2014; Bae et al., 2018), they pick up inputs from distinct sets of BC and AC types (Field et al., 2010; Helmstaedter et al., 2013). The selective connectivity with presynaptic neurons in the IPL is considered the foundation of the feature-selectivity of RGC pathways [e.g., (Roska and Werblin, 2001; Helmstaedter et al., 2013; Neumann et al., 2016; Yu et al., 2018)].

The response properties of an RGC type are typically determined by a hierarchy of mechanisms. For instance, the temporal response of an RGC to a light-step is shaped by presynaptic circuit components, such as glutamate receptor kinetics along the BC-RGC pathway (Awatramani and Slaughter, 2000; DeVries, 2000; Turner and Rieke, 2016; Yu et al., 2018), as well as by AC input (Nirenberg and Meister, 1997; Asari and Meister, 2012; Nikolaev et al., 2013; Jacoby et al., 2015; Cui et al., 2016; Franke et al., 2017). Moreover, the spatial receptive field (RF) is jointly formed by horizontal cells (Drinnenberg et al., 2018) and ACs (Diamond, 2017) in the outer and inner retina, respectively. In particular, AC circuits are very versatile “function modifiers”: For example, in the highly contrast-sensitive On alpha RGCs (Krieger et al., 2017), different AC circuits converge to provide On and Off inhibition to balance tonic excitatory drive from BCs (Park et al., 2018; Sawant et al., 2021); in On delayed RGCs, they provide a fast, excitatory surround through disinhibition (Mani and Schwartz, 2017).

In addition, RGC types differ in their expression of ion channels (Siegert et al., 2012; Rheaume et al., 2018; Tran et al., 2019) and how they are distributed across the cell [reviewed in (Van Hook et al., 2019)]. This ion channel complement, in combination with the RGC’s specific dendritic geometry, determines how synaptic input is integrated (Schachter et al., 2010; Poleg-Polsky and Diamond, 2011; Ran et al., 2020) and how the resulting signal is translated by the cell’s spike generator into action potentials on the optic nerve (Mobbs et al., 1992; Kim and Rieke, 2001, 2003; Raghuram et al., 2019; Werginz et al., 2020; Wienbar and Schwartz, 2022). Hence, RGCs “themselves” can significantly transform the input they receive from their circuits and thereby shape the retina’s output to the brain [reviewed in (Branco and Häusser, 2010; Stuart and Spruston, 2015; Tran-Van-Minh et al., 2015)].

The contribution of the aforementioned mechanisms to the RGC output varies among RGC types, yielding the diversity of feature representations, such as local edges (Levick, 1967; van Wyk et al., 2006; Zhang et al., 2012), approaching objects (Münch et al., 2009), “uniformity” (Levick, 1967; Jacoby et al., 2015; Tien et al., 2016), and direction of motion (Barlow et al., 1964). For this, intrinsic properties may play a pivotal role: For instance, On-Off direction-selective (DS) RGCs employ a range of mechanisms, including directionally tuned inhibitory and likely excitatory input from asymmetrically wired ACs, but their dendritic morphology and active channel distribution substantially contribute to these cells’ DS tuning [reviewed in (Borst and Euler, 2011; Mauss et al., 2017)].

Here, we study the role of a mechanism at the very end of the signal-shaping hierarchy, the encoding of the RGC membrane voltage into spike trains. We performed electrical single-cell recordings from a transient On RGC type that is EGFP-labelled in the transgenic *Igfbp5* mouse line (Siegert et al., 2009), which likely corresponds to G_18a_ [“ON trans”; (Baden et al., 2016)], “ON transient small RF” (Goetz et al., 2021), and “6 sn” (Bae et al., 2018). These cells display an unusual non-linear response behaviour in that they are sharply tuned to light stimuli with a high contrast while ignoring stimuli with low contrast (high contrast selectivity). Based on data from single-cell current analysis and computational modelling, we suggest that desensitisation of the spike generator of *Igfbp5-*positive transient On small (tOn-small) RGCs significantly contributes to both the transience of their light response and their selectivity for high contrasts.

## 2 Materials and methods

### 2.1 Animals and tissue preparation

The Tg(Igfbp5-EGFP)JE168Gsat transgenic mice of either sex were obtained from the Mutant Mouse Regional Resource Centre (MMRRC; University of California, Davis, CA, USA). In this transgenic mouse line, the EGFP reporter gene, followed by a polyadenylation sequence, was inserted into the BAC clone RP24-159O10 at the initiating ATG codon of the first coding exon of the Igfbp5 gene so that EGFP expression is driven by the regulatory sequences of the BAC gene (Gong et al., 2003). The resulting modified BAC (BX1812) was used to generate this transgenic mouse line (The Gene Expression Nervous System Atlas [GENSAT] Project, The Rockefeller University, New York, NY, USA), to which we refer in the following as “*Igfbp5*” line.

All procedures were performed in accordance with the law on animal protection issued by the German Federal Government (Tierschutzgesetz) and approved by the institutional animal welfare committee of the University of Tübingen or the MPI for Brain Research, Frankfurt/M. Mice of both genders (4–8 weeks of age) were housed under a standard 12 h day/night rhythm. For electrical recordings, mice were dark-adapted for ≥1 h before the experiment. The animals were then anesthetized with isoflurane (Baxter) and killed by cervical dislocation.

For immunostainings, the mouse eyes were dissected in cold 0.1 M phosphate buffer (PB), pH 7.4 and the posterior eyecups were immersion fixed in 4% paraformaldehyde (PFA) in PB for 15–30 min at room temperature. Following fixation, retinas were dissected from the eyecup, cryo-protected in graded sucrose solutions (10, 20, 30% w/v), and stored at –20°C in 30% sucrose until use. Retinal pieces were sectioned vertically at 16–20 μm using a cryostat.

For electrical recordings, the mouse eyes were enucleated and hemisected in carboxygenated (95% O_2_, 5% CO_2_) artificial cerebral spinal fluid (ACSF) solution, which contained (in mM): 125 NaCl, 2.5 KCl, 2 CaCl_2_, 1 MgCl_2_, 1.25 NaH_2_PO_4_, 26 NaHCO_3_, 0.5 L-glutamine, and 20 glucose; maintained at pH 7.4. After removal of the vitreous body, each retina was flat-mounted onto an Anodisc (#13, 0.2 um pore size, GE Healthcare, Maidstone, UK) with the ganglion cell layer facing up. The Anodisc with the tissue was then transferred to the recording chamber of a two-photon (2P) microscope or a Zeiss Axioscope (see below), where it was continuously perfused with carboxygenated ACSF. When using the two-photon microscope, ACSF contained 0.5–1 μM Sulforhodamine 101 (SR101, Invitrogen Steinheim, Germany) to reveal blood vessels and any damaged cells in the red fluorescence channel (see below). All procedures were carried out under very dim red (>650 nm) light.

### 2.2 Antibodies and immunohistochemistry

Cholinergic amacrine cells were labelled with goat anti-choline acetyltransferase (ChAT, 1:200; Chemicon), GABAergic amacrine cells with rabbit anti-GABA (1:2000; Sigma). A rat anti-glycine antibody labelled all glycinergic amacrine cells and ON cone bipolar cells (1:1000, kindly provided by David Pow, Brisbane, QLD, Australia). Rabbit anti-GFP was used to increase the EGFP signal (1:2000, Molecular Probes). The neurofilament marker SMI32 (mouse monoclonal, 1:1000; Sternberger Monoclonals) and a goat anti-osteopontin antibody (OPN, 1:1000; R&D Systems) were used to label alpha RGCs. Immunocytochemical labelling was performed using the indirect fluorescence method. Sections were incubated overnight with primary antibodies in 3% normal donkey serum (NDS), 0.5% Triton X-100, and 0.02% sodium azide in PB. After washing in PB, secondary antibodies were applied for 1 h. These were conjugated either to Cy3 (Dianova), or Alexa Fluor 488 (Invitrogen).

Confocal images were taken by using a Zeiss LSM 5 Pascal confocal microscope equipped with an argon and a HeNe laser. Images were taken with a Plan-Neofluar 40x/1.3 objective. Figures represent projections calculated from stacks of images with the LSM software or ImageJ (W.S. Rasband).^1^ Brightness and contrast of the final images were adjusted using Adobe Photoshop.

### 2.3 Single cell injection with DiI

For dye injections of BCs, enucleated eyes were transferred to oxygenated Ames medium (Sigma-Aldrich, Taufkirchen, Germany) and opened by an encircling cut. The retinas were dissected and embedded in 2% low melting agar (2-hydroxymethyl agarose, Sigma Aldrich), mounted on a vibratome (DSK Microslicer, DTK-1000, Ted Pella, Inc), and cut into 150 μm sections. After another 10 min in Ames Medium, sections were fixed in 4% PFA in PB at 4°C for 15 min. For injections with the fluorescent lipophilic tracer DiI (Molecular Probes) sharp microelectrodes were pulled from borosilicate glass tubing (Hilgenberg, Malsfeld, Germany) and filled with 0.5% DiI solution in 100% ethanol. The dye was injected into EGFP-labelled bipolar cells with 1 nA positive current for 3 min. For DiI injections of ACs and RGCs, retinal whole mounts were fixed in 4% PFA in PB for 15–30 min. After fixation, the tissue was kept in PB at 4°C overnight for the efficient diffusion of dye into the fine dendrites and axons. Filled cells were imaged with a confocal microscope.

### 2.4 Two-photon microscopy

We used a MOM-type two-photon microscope (designed by W. Denk, MPImF, Heidelberg; purchased from Science Products/Sutter Instruments, Novato, CA, USA). Both design and procedures were described previously (Euler et al., 2009). The system was equipped with a mode-locked Ti:Sapphire laser (MaiTai-HP DeepSee, Newport Spectra-Physics, Germany) tuned to 927 nm, two detection channels for fluorescence imaging (red, HQ 622 BP36; green, D 535 BP 50, or 520 BP 39; AHF, Tübingen, Germany) and a 20x objective (XLUMPlanFL, 0.95 NA, Olympus, Hamburg, Germany). The red fluorescence channel was used to visualize the retinal structure using SR101 staining (see above), the green channel to target EGFP-labelled *Igfbp5* RGCs.

### 2.5 Electrophysiology

In the *Igfbp5* line, two types of RGCs were targeted for single-cell electrical recordings: Non-fluorescent sOn-α cells, identified by their very large polygonal somata, and EGFP-labelled tOn-small RGCs, which possess medium-sized somata (∼15 μm in diameter). The identity of the recorded RGC type was confirmed after the recording based on the dendritic morphology. To avoid confounds related to morphological variability across the retina (Bleckert et al., 2014), the cells were collected (and recorded) from the ventral retina (∼0.8 mm from the optic disc).

For recording of light responses (current-clamp), electrodes (with resistances of 5–10 MΩ) contained (in mM): 120 K-gluconate, 5 NaCl, 10 KCl, 1 MgCl_2_, 1 EGTA, 10 HEPES, 2 Mg-ATP, and 0.5 Tris-GTP, adjusted to pH 7.2 using 1 M KOH. In addition, 4% Neurobiotin (Molecular Probes, Eugene, ORE, USA) and 0.2 mM SR101 were added to reveal the cell’s dendritic morphology. Membrane voltage was corrected for a liquid junction potential of ∼14 mV (Chang et al., 2013). Data were acquired using an Axoclamp-900A amplifier (Molecular Devices GmbH, San Jose, CA, USA) and digitized at 10 kHz. Experiments were carried out at 37°C. After the electrical recordings, the tissue was fixed, and cells were visualized by overnight incubation in 1:1000 Streptavidin-Alexa Fluor 594 (Invitrogen, Darmstadt, Germany). The RGC’s morphology (as image stack) was documented, and Z-projection images were made using ImageJ.

For voltage-clamp recordings, CdCl_2_ (100 μM) was added to the ACSF to block currents through voltage-gated Ca^2+^ channels. Electrodes (with resistances of 5–8 MΩ) contained (in mM): 120 Cs-gluconate, 1 CaCl_2_, 1 MgCl_2_, 10 Na-HEPES, 11 EGTA, 10 TEA-Cl, Sulforhodamine B (0.005%), adjusted to pH 7.2 with CsOH. Liquid junction potentials of 15 mV were corrected before the measurement with the pipette offset function of the amplifier (Schubert et al., 2008). Typically, the series resistance was around 7–18 MΩ for sON-a RGCs (13.4 ± 4.2 MΩ, mean ± SD, *n* = 18) and tOn-small RGCs (13.1 ± 3.4 MΩ, mean ± SD, *n* = 20). Cell capacitance was not compensated. Seal resistances >2 GΩ were routinely obtained. Cells were voltage-clamped at −90 mV and the different step protocols applied. All experiments were carried out at room temperature (20–22°C). Data were acquired using an Axopatch-200B amplifier (Molecular Devices, San Jose, CA, USA), digitized at 10 kHz using the pClamp software (Molecular Devices), and Bessel-filtered at 2 kHz.

### 2.6 Light stimulation

For light stimulation, we used a small reflective liquid-crystal-on-Silicon (LCoS) display (i-glasses; EST), coupled in the microscope’s optical path. The display was alternately illuminated by two bandpass-filtered (blue, 400 BP 10; green, 578 BP 10; AHF) LEDs, projecting spatio-temporally structured stimuli through the objective lens onto the retina (Euler et al., 2009). Stimulus intensity was measured using a calibrated photometer (Model 842-PE, 200–1100 nm, Newport) set to the respective centre wavelength of the LED filters. Cone photo-isomerization rates were calculated as described previously (Chang et al., 2013); all stimuli featured equal photo-isomerization rates for M- and S-opsin. Five stimulus protocols were used:

(a) a 200 μm-diameter bright spot flashed for 1 s,
(b) a 300 × 1000 μm bright bar moving at 500 μm/s in 8 directions,
(c) a 1 Hz sinusoidally modulated bright spot of varying diameter at 57% mean contrast,
(d) a 1 Hz sinusoidally modulated spot of varying contrasts, with the optimal spot diameter determined by stimulus (c),
(e) a flickering (white noise) spot, with the optimal spot diameter determined by stimulus (c); spot intensity was randomly chosen from a binary distribution for each frame (80 Hz refresh rate) and the mean intensity equal to background intensity.

For sinusoidally modulated stimuli (c, d), contrast was defined as the ratio of s.d. and mean intensity. The retina was always illuminated with a constant background in the (low) photopic range. In case of the protocols a and b, background illumination generated photo-isomerization rates (in 10^4^⋅P*s^–1^ per photoreceptor) of 1.2, 0.9, and 2.2, and in case of protocols c to e, 2.3, 2.2, and 4.8 for M-opsin, S-opsin and rhodopsin, respectively.

### 2.7 Data analysis

All electrophysiological data were analysed off-line using custom MATLAB scripts (Mathworks, Ismaning, Germany). To analyse tOn-small RGC responses to moving bars (protocol b), we defined the stimulus direction that generated most spikes (calculating the vector sum of the total spiking responses in 8 directions) as “preferred direction,” and calculated a direction selective index (*DSi*) as follows:

$$D\,S\,i=\frac{R_{P}-R_{N}}{R_{P}+R_{N}}$$

with preferred direction-response *R_P_* and null ( = opposite) direction response *R_N_*.

For the white noise stimulus (protocol e), we used the linear-non-linear (LN) cascade model described earlier (Chichilnisky, 2001; Kim and Rieke, 2001; Baccus and Meister, 2002; Field et al., 2010; Wang et al., 2011) to interpret RGC responses. This model consists of a linear filter that determines the cell’s temporal, chromatic and spatial sensitivities, as well as a “static” non-linearity that converts the filtered stimulus into a firing rate. In the time domain, the linear filter is proportional to the spike-triggered average stimulus (STA, the average stimulus preceding each spike) (Chichilnisky, 2001). Therefore, for LN models with identical linear filter but different non-linearities, spike-triggered average stimuli are identical up to a scale factor. We estimated the “static” non-linearity from the non-linear relationship between linear filtered responses (“generator signal”) and real responses.

Due to the limited refresh rate of white noise stimulus (80 Hz), we defined number of spikes in a time bin of △*t* = 12.5 ms (1/80 Hz) as the cell’s response, and STA was computed as the cross-correlation between response and stimulus. For graded signal, the cell’s response was defined as relative average voltage within each time bin (by subtracting the minimum voltage of the recorded trace).

We used the Difference-of-Gaussians (DOG) receptive field model in two dimensions to describe the spatial structure of the recorded RGCs. We assumed that the RF of each cell could be approximated by the weighted difference of two concentric Gaussian, one for the RF centre and one for the RF surround. A non-linear function was included into the model to convert this weighted sum into the firing rate (Yin et al., 2009). An RGC’s response *R* to a spot stimulus centred in the RF and with diameter *D* and contrast *c* was calculated as follows:

$$R=f\,\left(\iint_{x^{2}+y^{2}\leq D^{2}/4}\left(A_{C}\,\frac{1}{2\,\pi \,\sigma_{C}^{2}}\,e^{\frac{x^{2}+y^{2}}{2\,\sigma_{c}^{2}}}-A_{S}\,\frac{1}{2\,\pi \,\sigma_{S}^{2}}\,e^{\frac{x^{2}+y^{2}}{2\,\sigma_{s}^{2}}}\right)\,c\,dx\,dy\right)$$

with the non-linear function *f*, amplitude (*A_C_*) and spatial extent (σ_*C*_) of the RF centre, as well as amplitude (*A_C_*) and spatial extent (σ_*S*_) of the RF surround.

The non-linear function *f* was determined using the same RGC’s responses to spots with fixed diameter but varying contrasts, fitted by the Naka-Rushton Equation (Naka and Rushton, 1966):

$$R\,\left(c\,o\,n\,t\,r\,a\,s\,t\right)=R_{m\,a\,x}\,\frac{c^{n}}{c^{n}+c_{1/2}^{n}}$$

The other four parameters (*A_C_*, *A_S_*, σ_*C*_, σ_*S*_) were determined by fitting the model to area summation data using a numerical search.

### 2.8 Computational modelling

To explain our experimental data, we constructed a one-compartment Hodgkin-Huxley model (Dayan and Abbott, 2005):

$$\begin{array}{c} c_{m}\,\frac{d\,V}{d\,t}=-{\bar{g}}_{N\,a}\cdot m^{3}\cdot h\cdot \left(V-E_{N\,a}\right)-{\bar{g}}_{K}\cdot n^{4}\cdot \left(V-E_{K}\right)\\ -{\bar{g}}_{L}\cdot \left(V-E_{L}\right)+\frac{I_{e}+I_{n}}{A} \end{array}$$

with the specific membrane capacitance *c_m_* = 10 nF/mm^2^, the voltage across the cell membrane (*V*) in mV, the maximal conductance for transient Na^+^ current (${\bar{g}}_{N\,a}$), delayed-rectifier *K*^+^ current and a leakage current (${\bar{g}}_{N\,a}$ = 1.2 mS/mm^2^; ${\bar{g}}_{K}$ = 0.05 mS/mm^2^;${\bar{g}}_{L}$ = 0.003 mS/mm^2^), the reversal potentials for the three currents (*E*_*Na*_ = 50 mV; *E_K_* = −76 mV; *E_L_* = −70 mV), the area of the cell membrane (*A* = 0.0013 mm^2^), the injected current (*I_e_*), and a noise component (*I_n_*) with a 1/*f* power spectrum (pink noise). *I_e_* is the estimated response to a sequence of binary white noise, using the linear filter and static non-linearity determined from graded voltage responses. In brief, the linear temporal filter was estimated by computing the spike-triggered average (STA). The inner product between the STA and the actual stimuli yielded a generator signal, and the relationship between the actual response and the generator signal was used to estimate the static non-linearity (Chichilnisky, 2001). *I_e_* was computed by first convolving the light stimuli with the linear filter and then passing the results through the non-linearity. *I_e_* were current steps with varying amplitudes. The gating variables were updated as following:

$$\text{d}\,n/\text{d}\,t=\,\alpha_{n}\,\left(1-n\right)-\beta_{n}\,n$$

$$\text{d}\,m/\text{d}\,t=\,\alpha_{m}\,\left(1-m\right)-\beta_{m}\,m$$

$$\text{d}\,h/\text{d}\,t=\,\alpha_{h}\,\left(1-h\right)-\beta_{h}\,h$$

As rate constants for *m*, *h*, and *n* we used (Hodgkin and Huxley, 1952):

$$\begin{array}{cc} \alpha_{m}=0.1\,\frac{V+40}{1-e^{-0.1\,\left(V+40\right)}}\; & \beta_{m}=4\,e^{-0.0556\,\left(V+65\right)}\\ \alpha_{h}=0.07\,e^{-0.05\,\left(V+65\right)}\; & \beta_{h}=\frac{1}{1-e^{3-0.1\,\left(V+65\right)}}\\ \alpha_{n}=0.01\,\frac{V+55}{1-e^{-0.1\,\left(V+55\right)}}\; & \beta_{n}=0.125\,e^{-0.0125\,\left(V+65\right)} \end{array}$$

After each spike, the voltage dependences of the rate constants (α and β) for *m* and *h* were increased by 1.55 mV for the sensitising model (i.e., tOn-small RGCs) and by 0.01 mV for the non-desensitising case (i.e., sON-α RGCs). For example, after an action potential, $\alpha_{m}=0.1\,\frac{V-d\,V+40}{1-e^{-0.1\,\left(V-d\,V+40\right)}}$ changed with *dV* = 1.55 mV for the desensitising model and 0.01 mV for the non-desensitising model. The recovery from this shift in voltage dependence followed an exponential function with a time constant of 5 s.

### 2.9 Space-clamp modelling

Based on the differences in neuronal morphology, we built a ball-and-stick model for each cell type (Ran et al., 2020). To get precise measurements of dendritic lengths and radii for sOn-α and tOn-small cells, we extracted this information from published morphologies of 8 w (*n* = 4) and 6 sn (*n* = 6) cells, reconstructed from a mouse retina electron microscopy data set.^2^ Soma diameters were estimated from our data [∼20 μm for sOn-α (8 w) cells and ∼15 μm for tOn-small (6 sn) cells]. We used the median of these values in our model, which was implemented in NEURON.^3^ Each dendritic portion was further divided into multiple segments with a maximal length of 7 μm. The 1D model can be characterized by the cable equation:

$$\frac{\text{d}}{4\,{\text{r}}_{\text{a}}}\,\frac{\partial^{2}\text{V}}{\partial {\text{x}}^{2}}={\text{C}}_{\text{m}}\,\frac{\partial \text{V}}{\partial \text{t}}+{\text{I}}_{\text{ion}}-{\text{I}}_{\text{comp}}$$

where V is the voltage across the cell membrane, x is the distance along the cable, d is the dendritic diameter, r_a_ is the intracellular resistivity, C_m_ is the specific membrane capacitance, I_comp_ is the compensated current to clamp the voltage at the soma according to the command voltage, I_ion_ represents the sum of the sodium current and the leak current, the current dynamics are described following Fohlmeister and Miller (1997) as:

$${\text{I}}_{\text{ion}}={\text{I}}_{\text{Na}}+{\text{I}}_{\text{Leak}}={\bar{\text{g}}}_{\text{Na}}\cdot {\text{m}}^{3}\cdot \text{h}\cdot \left(\text{V}-{\text{E}}_{\text{Na}}\right)+{\bar{\text{g}}}_{\text{L}}\cdot \left(\text{V}-{\text{E}}_{\text{L}}\right)$$

The elicited potential of each dendritic segment was recorded. Model parameters, channel conductances, and dendritic parameters are shown in Tables 1–3, respectively.

**TABLE 1:** Model parameters.

Parameters	Values
Temperature	*T* = 32°C
Intracellular axial resistivity	*R*_*a*_ = 110 Ωcm
Specific membrane resistance	*R*_*m*_ = 15,000 Ωcm^2^
Specific membrane capacitance	*C*_*m*_ = 1 μF cm^–2^

**TABLE 2:** Reference distribution of ion channels in cellular compartments.

Channel type	Conductance in soma (S cm^–2^)	Conductance in dendrites (S cm^–2^)
g_*Na*_	0.08	0.025
g_*Ca*_	0	0
g_*K*_	0	0
g_*K,A*_	0	0
g_*K,Ca*_	0	0

**TABLE 3:** Dendrite morphology parameters.

Branch order	Branch length in sOn-α cells (μ m)	Branch diameter in sOn-α cells (μ m)	Branch length in tOn-small cells (μ m)	Branch diameter in tOn-small cells (μ m)
Branch 01	14.91	0.72	11.83	0.7
Branch 02	22.65	0.52	7.47	0.59
Branch 03	36.84	0.5	5.03	0.46
Branch 04	30.98	0.43	5.84	0.39
Branch 05	40.81	0.44	5.04	0.36
Branch 06	32.22	0.42	4.82	0.34
Branch 07	53.79	0.43	6.8	0.36
Branch 08	38.92	0.43	5.95	0.36
Branch 09	18.24	0.36	5.2	0.36
Branch 10			5.75	0.34
Branch 11			5.12	0.31
Branch 12			5.62	0.34
Branch 13			5.6	0.31
Branch 14			7.09	0.33
Branch 15			6.62	0.33
Branch 16			7.51	0.32
Branch 17			4.13	0.33
Branch 18			7.51	0.28
Branch 19			7.73	0.32
Branch 20			5.71	0.25

### 2.10 Statistics

Data presentation and statistical analysis were performed using Matlab or in the R programming language. The Wilcoxon signed-rank test was used to determine statistical significance between different conditions. Significance was defined as[: *p* > 0.05 = non-significant (ns), *p* < 0.05 = *, *p* < 0.01 = **]. Mean values in text and figures are given as mean ± standard error of the mean (SEM).

## 3 Results

### 3.1 EGFP-expressing neurons in the Igfbp5 mouse retina

**FIGURE 1:**
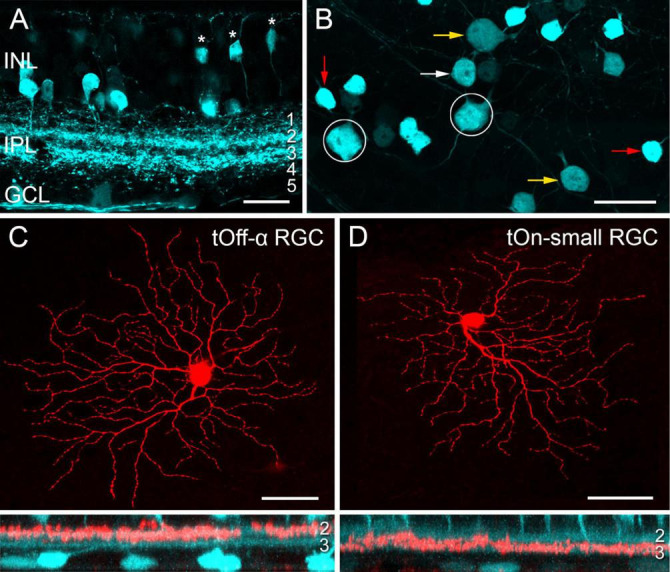
EGFP-labelled cells in the *Igfbp5* transgenic mouse line. **(A)** EGFP expression in a vertical section of the *Igfbp5* retina, with EGFP expressed in bipolar cells (BCs, asterisks) and amacrine cells (ACs) in the inner nuclear layer (INL), as well as in displaced amacrine cells and retinal ganglion cells (RGCs) in the ganglion cell layer (GCL). EGFP-labelled processes extend along sublaminae 2 and 3 of the inner plexiform layer (IPL). **(B)** Whole-mount showing mosaic of EGFP-expressing somata in the GCL. Examples of putative displaced ACs (red arrows), Off RGCs with medium-sized (white arrows) and large (yellow arrow) somata, and On RGCs (circles) indicated. Examples of EGFP-positive RGCs: tOff-α RGC **(C)**, and tOn-small RGC **(D)** filled with Neurobiotin during electrical recording. Below each panel, z-projections of the cell’s dendritic arborisation. Scale bars: **(A)**, 20 μm; **(B)**, 25 μm; **(C)** and **(D)**, 50 μm.

Transgenic mice, in which specific RGC types are fluorescently labelled, greatly facilitate investigating RGC function (e.g., Münch et al., 2009; Bleckert et al., 2014; Rousso et al., 2016; Yao et al., 2018). While screening the Gene Expression Nervous System Atlas (GENSAT) database of transgenic mice for selective lines (Siegert et al., 2009), we were struck by the very distinctive pattern of retinal EGFP expression in the *Igfbp5* (insulin-like growth factor-binding protein 5) mouse line (Figures 1A, B). In the *Igfbp5* retina, the EGFP is expressed in two prominent, equally thick bands of processes along IPL sublaminae 2 and 3 (Figure 1A and Supplementary Figures 1A–C), extending just in between the two choline-acetyltransferase (ChAT) -positive bands (Supplementary Figures 1D, E). This pattern is reminiscent of the glypho (glycogen phosphorylase) staining in the macaque monkey retina (Majumdar et al., 2008). We detected EGFP in a subset of BCs and ACs in the inner nuclear layer (INL; Figure 1A; for details, see Supplementary Figures 1A–D), as well as in some RGCs and displaced ACs in the ganglion cell layer (GCL; Figure 1B).

In the GCL, four cell types could be distinguished based on their soma size and labelling intensity (Figure 1B): very bright cells with small somata (soma diameter: 9.1 ± 0.5 μm, mean ± s.d., *n* = 29), dimmer cells with medium-sized (12.0 ± 0.5 μm, *n* = 24) or larger somata (14.8 ± 0.9 μm, *n* = 34), and very dim cells with large somata (17.5 ± 0.7 μm, *n* = 14).

Dye-injections (*n* = 40) revealed that the small, brightly labelled cells were likely displaced wide-field ACs (Lin and Masland, 2006): They featured 6–10 primary dendrites that sometimes bifurcated close to the soma (∼14 dendrites/cell in total), rarely crossed each other and narrowly stratified in IPL sublamina 3 (Supplementary Figures 1F, 2A). The dendritic fields of neighbouring cells overlapped frequently, resulting in dense retinal coverage (Supplementary Figures 2B, C). These putative ACs closely resembled glypho-positive On ACs in the macaque (Majumdar et al., 2008).

The three other *lgfbp5-*positive cells in the GCL were monostratified RGCs (Figures 1C, D and Supplementary Figures 3A–C) with their dendrites in either one of the two EGFP bands (*cf*. Table 4). The cells with the largest, dimmest somata were transient Off-alpha (tOff-α) RGCs (Figures 1B, C and Supplementary Figure 3B; van Wyk et al., 2009; Krieger et al., 2017; Ran et al., 2020), as confirmed by immunolabeling (Supplementary Figures 3E–H) for markers such as SMI32 (Supplementary Figures 3E, F), which strongly labels sustained On alpha (sOn-α) and tOff-α cells in mouse (Coombs et al., 2006; Bleckert et al., 2014). The remaining two labelled cell populations had dendritic arbour diameters of around 200 μm.

**TABLE 4:** Igfbp5-positive RGCs and their presumed morphological and genetic counterparts in earlier mouse studies.

RGC type in *lgfbp5* line	IPL depth	RGC types in recent studies							
		**Bae et al., 2018**	**Kong et al., 2005**	**Farrow et al., 2013**	**Sümbül et al., 2014**	**Sun et al., 2002**	**Goetz et al., 2021**	**Baden et al., 2016**	**Tran et al., 2019**
Off (medium soma, bright)	S2	4i or 4on	cluster 3	PV4	CB2	RGB1 or outer RGB3	OFF transient small RF	G9-OFF T alpha mini*	Tbr1-S2, cluster 21
On (large soma, bright)	S3	6sn	cluster 2	PV2	cluster X	inner RGB3	ON transient small RF	G18a-ON transient or G23-ON alpha mini*	–
Trans. Off -α (largest soma, dim)	S2	4ow	–	PV5	W7a	A2 outer	OFF transient alpha	G8-OFF T alpha	Alpha OFF T, cluster 45

Missing entries indicate that type assignment is unclear.

*As discussed in Tran et al. (2019).

The *lgfbp5-*positive cells with medium-sized somata were presumably Off cells, as their dendrites stratified in sublamina 2; their morphology (Supplementary Figure 3A) resembled that of “4i” or “4on” cells described in Bae et al. (2018), cluster 3 (Kong et al., 2005), PV4 (Farrow et al., 2013), CB2 (Sümbül et al., 2014), and RGB1 or “outer” RGB3 cells (Sun et al., 2002).

**FIGURE 2:**
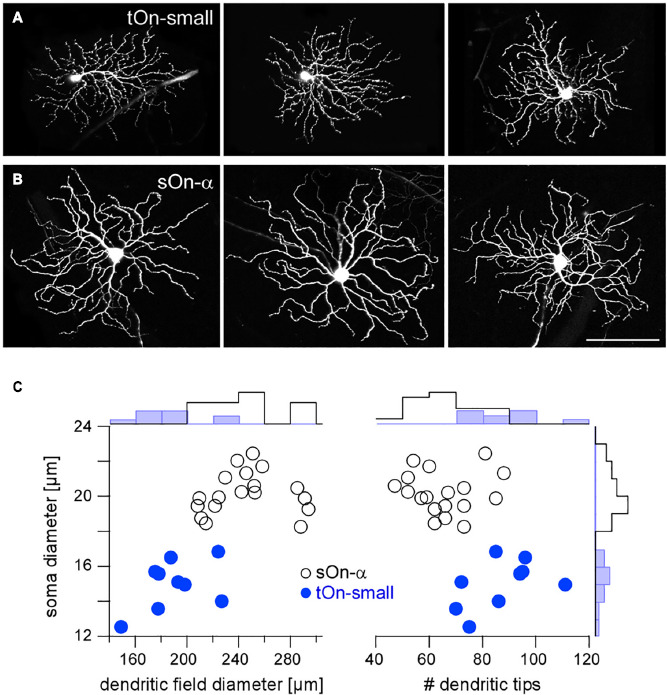
*Igfbp5*-positive tOn-small ganglion cells differ morphologically from sustained sOn-α cells. Examples of dye-injected RGCs: three tOn-small **(A)** and three sOn-α cells **(B)**. To avoid confounds related to morphological variability across the retina (Bleckert et al., 2014), the cells were collected (and recorded) from the ventral retina (∼0.8 mm from the optic disc). **(C)** Soma diameter vs. dendritic field diameter (left) and vs. number of dendritic tips (right) for the two RGC types, with distribution histograms at the sides. Scale bar: **(A,B)**, 100 μm. For a more detailed overview of *Igfbp5*-positive RGCs and comparison with primate retina see Supplementary Figure 3.

The mouse *lgfbp5-*positive RGCs with the larger somata (Figures 1D, 2A, C and Supplementary Figure 3C) were presumably On cells as they stratified in sublamina 3; their morphology resembled that of “6sn” (Bae et al., 2018), “ON transient small RF” (Goetz et al., 2021), cluster 2 (Kong et al., 2005), PV2 (Farrow et al., 2013), “cluster X” (Sümbül et al., 2014), and “inner” RGB3 cells (Sun et al., 2002). In the transcriptomics study by Tran et al. (2019), this RGC type remained elusive. For simplicity, we refer to the *lgfbp5*-positive On RGCs in the following as transient On small (tOn-small) cells. Mouse transient On-alpha (tOn-α) RGCs stratify at the same level as the tOn-small cells but have a slightly larger dendritic tree (Goetz et al., 2021) and are osteopontin-positive (Krieger et al., 2017). We think that the tOn-small RGCs are good candidates for being homologous to primate parasol cells (for discussion see also Hahn et al., 2023; Supplementary Figure 3I).

### 3.2 tOn-small RGC responses are highly transient

When we characterized light-evoked signals in tOn-small cells using two-photon-guided electrical recordings (see section “2 Materials and methods”), we were intrigued by their very transient responses—with the spike rate increasing almost instantaneously at light-onset but then quickly dropping to zero within ∼200 ms and no response at light-offset (Supplementary Figure 4A). As such transient responses were described in a type of direction-selective On RGC in rabbit (Kanjhan and Sivyer, 2010), we recorded tOn-small cell responses to a moving bar stimulus, but did not find any substantial directional tuning in these cells (Supplementary Figure 4B; DSi = 0.043 ± 0.019, *n* = 5 cells).

In the following, we studied the mechanisms underlying the characteristic transient responses of tOn-small cells in the mouse retina (Figure 2A). For comparison, we recorded the well-described sOn-α RGCs (Peichl et al., 1987), which are known for their sustained responses and their high sensitivity for small contrast changes (high contrast sensitivity) (Pang et al., 2003; van Wyk et al., 2009; Schwartz et al., 2012; Bleckert et al., 2014; Krieger et al., 2017; Figure 2B). Morphologically, the two RGC types could be distinguished easily, with tOn-small cells having smaller somata and dendritic fields, but more dendritic tips than sOn-α cells (Figure 2C).

### 3.3 tOn-small cells encode selectively high-contrast signals

**FIGURE 3:**
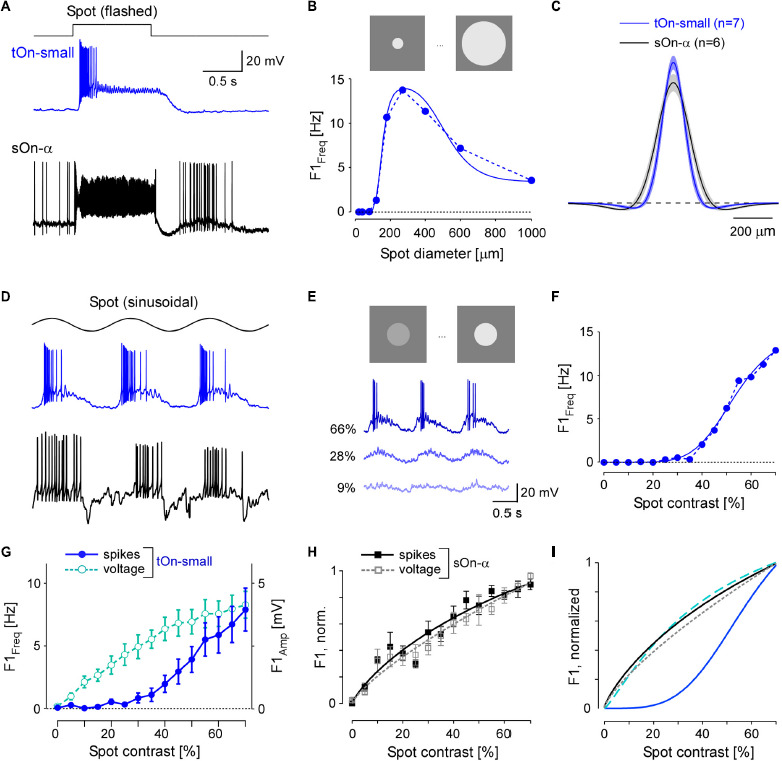
tOn-small ganglion cells have smaller receptive fields (RFs) and higher contrast thresholds compared to sOn-α cells. **(A)** Electrically recorded voltage responses of a tOn-small (blue) and a sOn-α (black) to a 1-s flashed spot (200 μm in diameter). **(B)** Fundamental (F1) spiking response (F1 Freq in Hz) as a function of spot diameter (1 Hz, 57% mean contrast) for the same tOn-small cell as in **(A)** (dotted blue line). Data fit with Difference-of-Gaussians (DOG) receptive field model (solid blue line). **(C)** Mean and SEM of estimated RF profiles, using difference-of-Gaussians (DOG) RF model for *n* = 7 tOn-small and *n* = 6 sOn-α cells (see section “2 Materials and methods”). **(D)** Responses of the same cells as in **(A)** to a 1-Hz sinusoidal stimulus (270 μm in diameter). **(E)** Representative responses of a tOn-small cell for different contrasts. **(F)** F1 Freq as a function of stimulus contrast (D1; 270 μm diameter spot; data fit with Naka-Rushton equation; see section “2 Materials and methods”) for the same tOn-small cell as in **(E)**. **(G)** Response-contrast functions for tOn-small cells (blue, spikes, *n* = 7 cells; cyan, voltage, *n* = 3 cells). **(H)** Normalized response-contrast curves for an additional set of sOn-α cells (black, spikes, *n* = 6 cells; grey, voltage, *n* = 6 cells; Naka-Rushton fits). **(I)** Normalized response-contrast curves [dataset from **(G,H)**; Naka-Rushton fits]. Symbols and error bars represent mean and SEM, respectively. For responses of tOn-small cells to moving bar stimuli see Supplementary Figure 4.

First, we studied the spatial RF organisation of tOn-small cells using spot stimuli with varying diameters centred on the soma and sinusoidally modulated at 1 Hz (Figures 3A, B), in comparison to sOn-α cells. Using spectral analysis, we then calculated the amplitude of the cells’ fundamental response component (see section “2 Materials and methods”). With increasing spot diameter, amplitudes first increased and then declined (Figure 3B), indicative of centre-surround antagonism. To quantify the spatial RFs, we used a Difference-of-Gaussians (DOG) model to interpret the recorded area summation data (see section “2 Materials and methods”). Consistent with their larger dendritic field diameters (Figure 2C), sOn-α cells had larger RF centres than tOn-small cells (Figure 3C).

In the DOG model, we included a static-non-linearity to convert the weighted stimulus into a firing rate. Because the area summation data by itself was not sufficient to characterize this non-linearity, we also presented spots with fixed diameter but with different contrasts (Figure 3D). Here, we used the spot diameter (270 μm) that elicited the maximal spiking response. Other than sOn-α cells, tOn-small cells were insensitive to weak stimuli: Sinusoidally modulated spots with contrasts lower than 40% hardly evoked any spiking response (Figures 3E, F).

We also analysed the graded voltage responses recorded in the whole-cell current-clamp mode (with spikes digitally removed, see section “2 Materials and methods”) and found that the strong threshold-like behaviour was not reflected in the graded response: tOn-small cells showed a detectable depolarization even to the smallest contrast tested (5%, Figure 3G, cyan circles vs. blue circles), resulting in a contrast sensitivity curve similar to that for the sOn-α cells’ spiking response (Figure 3H; black squares). Note that sOn-α cells displayed a very similar contrast sensitivity curve for spikes and graded voltage (Figure 3H; filled black vs. open grey squares). Together, this suggests that the conversion of voltage into a spiking response differs between tOn-small and sOn-α cells (Figure 3I). A possible explanation may be a larger difference between resting potential and spiking threshold in tOn-small vs. sOn-α cells. While the resting potential (V_*rest*_) of tOn-small cells (−66.8 ± 3.4 mV; *n* = 6) indeed was slightly more hyperpolarized than that of sOn-α cells (−64.8 ± 3.3 mV; *n* = 6), the difference seems too small to explain the observed difference in contrast sensitivity alone. Note that the estimate of the resting potentials may not be accurate due to the perturbation of the intracellular milieu in whole-cell experiments. We tried to mitigate this problem by reading the resting potential as soon as the whole-cell recording is established. We also estimated the difference between the spiking threshold and the resting potential and found that the results of the tOn-small cells (12.2 ± 3.5 mV; *n* = 3) were only slightly higher than those of sOn-α cells (9.3 ± 2.3 mV; *n* = 4).

**FIGURE 4:**
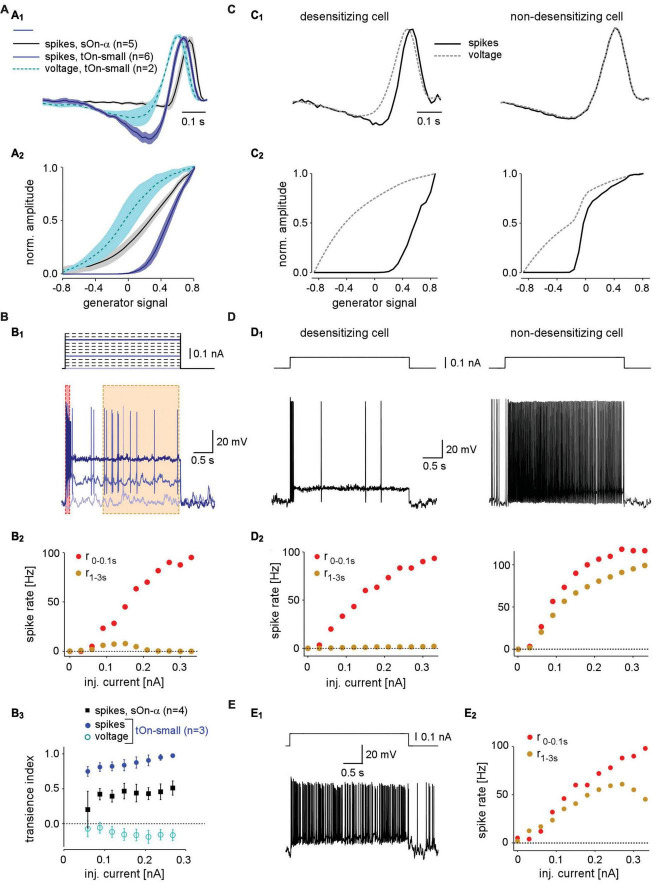
Spike desensitisation in tOn-small ganglion cells accounts for their biphasic temporal filter and a high contrast-selective threshold. **(A)** Temporal linear filter **(A_1_)** and static non-linearity **(A_2_)** estimated using a white noise stimulus (see section “2 Materials and methods”) for tOn-small (blue, spikes, *n* = 6 cells; cyan, graded voltage responses, *n* = 2 cells) and sOn-α cells (black, spikes, *n* = 5 cells). **(B)** Electrically recorded voltage responses of a tOn-small cell [**(B_1_)**, bottom, resting potential V_*rest*_ = –66 mV] in response to current injections of varying amplitude [**(B_1_)**, **top**]. **(B_2_)**, Firing rate of the same cell right after current injection onset (0–0.1 s, red) and at steady-state (1–3 s, brown), as functions of injected current [for time windows, see **(B_1_)**]. **(B_3_)** Relationship between spike desensitisation and injected current for tOn-small (blue, spikes; cyan, graded voltage responses, *n* = 3) and sOn-α cells (black, spikes, *n* = 4). Spike desensitisation was quantified as transience index (*Ti*_*spike*_1−(*r*_1−3*s*_/*r*_0−0.1*s*_) or *Ti*_*V*_1−(*V*_1−3*s*_/*V*_0−0.1*s*_), with spike rate *r* and baseline-corrected graded potential *V*, respectively). Symbols and error bars represent mean and SEM, respectively. **(C)** Temporal linear filter **(C_1_)** and static non-linearity **(C_2_)** estimated using simulated responses of two modelled RGCs (left: with strong spike desensitisation; right: with weak spike desensitisation) to a white noise stimulus (for details, see text). **(D)** Voltage responses of the two modelled cells to current injection of 0.1 nA **(D_1_)** and their firing rates as functions of injected current [**(D_2_)**, analogous to recording in **(B_2_)**]. **(E)** Voltage recording of a sOn-α cell (V_*rest*_ = –64 mV) in response to current injections of 0.1 nA **(E_1_)** and its firing rate as a function of injected current [**(E_2_)**, analogous to **(B_2_)**].

The difference in contrast sensitivity between spiking and graded voltage responses in tOn-small cells is striking. Related to this was our finding that when stimulated with a bright flash, the cells displayed a sustained graded voltage response—in contrast to the transient nature of their spiking response (Figure 3A). We therefore studied temporal processing in tOn-small (and, for comparison, sOn-α) cells with a homogeneous white-noise stimulus (see section “2 Materials and methods”), using again the optimal spot diameter. First, we calculated the spike triggered average (STA) as an estimate of the cell’s temporal linear filter (Chichilnisky, 2001). tOn-small cells had highly biphasic filters, with an upward-pointing lobe (On-lobe) close to the time of spike and an almost similarly strong lobe pointing downward (Off-lobe) further away (Figure 4A1; blue curve). We quantified the filter’s biphasic nature by calculating the ratio between Off- and On-lobe peak (Chander and Chichilnisky, 2001), yielding a biphasicity index (*B*_*i*_) of 0.60 ± 0.19 (*n* = 6) for the spike response of tOn-small cells. In contrast, the linear filter estimated from their graded voltage response (*B*_*i*_ = 0.28 ± 0.17, *n* = 2) and the STA-derived linear filters of sOn-α cells (*B*_*i*_ = 0.15 ± 0.09, *n* = 5) were more monophasic (Figure 4A1; dashed cyan and black curve, respectively).

The difference between linear filters estimated from spike vs. voltage responses indicates that the non-linearity underlying spike generation in tOn-small cells is not a “trivial” static non-linearity, i.e., a simple non-linear function whose output only depends on the present value of input. Because the linear filter estimation is expected to be resistant to a static non-linearity (Chichilnisky, 2001), the involvement of a static non-linearity should result in similar filters—independent of the response modality used (spikes vs. graded voltage). To characterize the non-linearity in tOn-small cells, we calculated the relationship between the linear response (“generator signal” = the convolution of estimated linear filter and stimulus) and the recorded response to the white noise stimuli. The resulting non-linear function for the spike responses of tOn-small cells had a much higher threshold than that for their voltage responses and the spike responses of sOn-α cells (Figure 4A2).

Many response features of RGCs, including temporal properties and contrasts sensitivity, are shaped by synaptic interactions (see sections also “1 Introduction and 4 Discussion”). However, the clear differences in transiency and contrast sensitivity between spiking and graded voltage responses observed in tOn-small (but not sOn-α) RGCs point at a contribution of the tOn-small cells’ intrinsic spike generator (Li et al., 2022). Therefore, in the following we focused on this final step in retinal signal transformation.

### 3.4 Spike generation in tOn-small RGCs is readily desensitised by constant current injection

The comparison between spike and graded voltage responses revealed two distinct features of spike generation in tOn-small cells: (i) the high-threshold non-linearity and (ii) the pronounced biphasicity of the linear filter. The first feature could arise simply from a spike threshold that is substantially higher than the resting potential. The second feature implies more complicated non-linearities, such as spike desensitisation (Goldin, 1999). We tested the spike generator by injecting constant current of different amplitudes into the cells while recording their voltage responses (Figure 4B1). We found that for tOn-small cells, injection of a large current elicited a burst of spikes at injection onset but then spiking stopped rapidly (Figure 4B1, dark blue trace). The initial spike frequency (time window: 0–0.1 s) scaled almost linearly with the amplitude of injected current (Figure 4B2, red symbols), whereas the steady-state spike frequency remained as low as ∼10 spikes/s or less (Figure 4B2, orange symbols). As a result, transiency of the spike response increased with stimulus strength, as indicated by the increasing ratio between initial spiking response and steady state (Figure 4B3). This increase was absent or less pronounced in graded voltage responses of tOn-small cells and spiking responses of sOn-α cells, respectively. Together, this points at strong desensitisation of the spike generator in tOn-small cells.

### 3.5 Spike desensitisation model mimics response properties of tOn-small RGCs

We constructed a simple model to explore how varying levels of spike desensitisation affect the signal encoding properties of desensitising and non-desensitizing cells, such as tOn-small and sOn-α cells, respectively (see section “2 Materials and methods”). We modelled responses to the white-noise stimulus by calculating the amplitude of the input current to the cell using a simple linear-non-linear (LN) model. First, for the desensitising case (Figures 4C1, C2; left), we estimated the linear filter and non-linearity from graded voltage responses measured in tOn-small RGCs (Figures 4A1, A2). Using the voltage output of the modelled cell, we then estimated linear filter and non-linearity for the spike responses. We found that the spike response-derived linear filter was more biphasic than that based on the graded voltage response, very similar to what we measured in tOn-small cells (cf. Figures 4A1, A2). For the non-desensitising case (Figures 4C1, C2, right), the two linear filters were nearly identical. In both model cells, the non-linearity for spike responses exhibited a higher threshold than that for graded voltage responses, however, only the threshold in the desensitising cell was higher than the linear response to 0% contrast (generator signal = 0; Figure 4C2; cf. panel Figure 4A2). Therefore, low-contrast stimuli (generator signal ∼0) evoked only sub-threshold responses in the desensitising model cell—very similar to the responses recorded in tOn-small cells (cf. Figure 3E).

We also used current steps with different amplitudes as model input (Figure 4D). As expected, the desensitising model cell displayed little or no spike response at the steady state (1–3 s) of the current step (Figure 4D, left), very similar to the responses recorded in tOn-small RGCs (Figure 4B1). In contrast, the steady-state response of the non-desensitising model cell was nearly as strong as its response at the current injection onset (0–0.1 s; *r*_2−3*s*_*r*0−0.1*s*) for each current amplitude (Figure 4D, right), very similar to the recorded response of sOn-α cells (Figure 4E).

In conclusion, our model supports that spike desensitisation, and a high spiking threshold may contribute to two distinct response features of tOn-small RGCs: their strong transience and their selectivity for high contrasts. Note that our simple desensitising model generates responses very similar to those of tOn-small cells, whereas the match between the non-desensitising model and sOn-α cells was not as good: The non-linearity is “steeper” in the non-desensitising model cell than that estimated from sOn-α cell recordings (Figure 4C2 right vs. Figure 4A2). This discrepancy suggests that a sOn-α cell model requires additional properties, such as, for example, a higher noise level in the membrane potential, which can result in a smoother non-linearity (Anderson et al., 2000). Although our model is rather simple and does not take the detailed desensitization states of the voltage-gated channels into account, it supports that desensitization of voltage-gated sodium channels (VGSCs) can explain the differences we observed in two types of RGCs.

### 3.6 Voltage-gated Na^+^ channels in tOn-small RGCs are high-threshold activated

**FIGURE 5:**
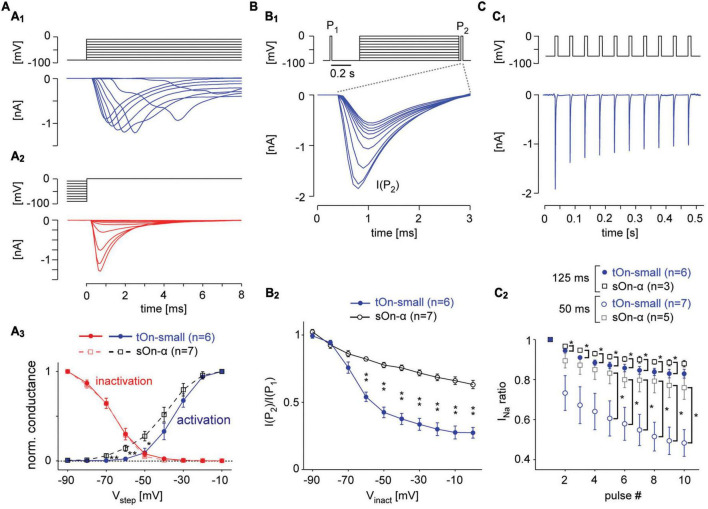
Voltage-gated Na^+^ channels in tOn-small cells desensitise strongly. **(A)** Voltage-clamp recording of a tON-small cell to step protocols probing activation **(A_1_)** and inactivation **(A_2_)** of voltage-gated Na^+^ channels [VGSCs; **(A_1_)**, from –90 mV for 1 s to different voltages (–90 to –10 mV); **(A_2_)**, from different potentials (–90 to –10 mV) for 1 s to 0 mV; ΔV = 10 mV for both protocols]. **(A_3_)** Normalized conductance as functions of step voltage (V_*step*_) for channel activation (squares) and inactivation (circles) in tOn-small (blue, *n* = 6) and sOn-α cells (black, *n* = 7). **(B)** Probing inactivation of VGSCs with 3-ms test pulses (to 0 mV) delivered before (P_1_) and after (P_2_) the 1-s inactivating steps [**(B_1_)**, top; –90 to 0 V, ΔV = 10 mV, followed by a hyperpolarising step for 20 ms to –90 mV]. Na^+^ currents elicited by the 2nd test pulse (P_2_) in a tOn-small cell [**(B_1_)**, bottom]. **(B_2_)** Ratio of Na^+^ current amplitudes elicited by the two test pulses [cf. **(B_1_)**] plotted against inactivating voltage. **(C)** Probing “inactivation sensitivity” using sequences of 10-ms depolarizing voltage pulses (–70 to 0 mV). **(C_1_)** Na^2+^ currents elicited in a tOn-small cell by a 20-Hz sequence. **(C_2_)** Peak Na^+^ currents as a function of pulse number for 20 (Δt = 50 ms) and 8 Hz (Δt = 125 ms blue circles and black squares for tOn-small and sOn-α cells, respectively). Peak Na^+^ currents were normalized to the amplitude evoked by the first pulse. Data from tOn-small (circles, *n* = 7) and sOn-α cells (squares, *n* = 5) for 20 Hz pulses, and tOn-small (circles, *n* = 6) and sOn-α cells (squares, *n* = 3) for 8 Hz pulses. Wilcoxon rank sum test was used for determining statistical significance (*p < 0.05, **p < 0.01) between different conditions in **(A_3_,B_2_,C_2_)**.

**FIGURE 6:**
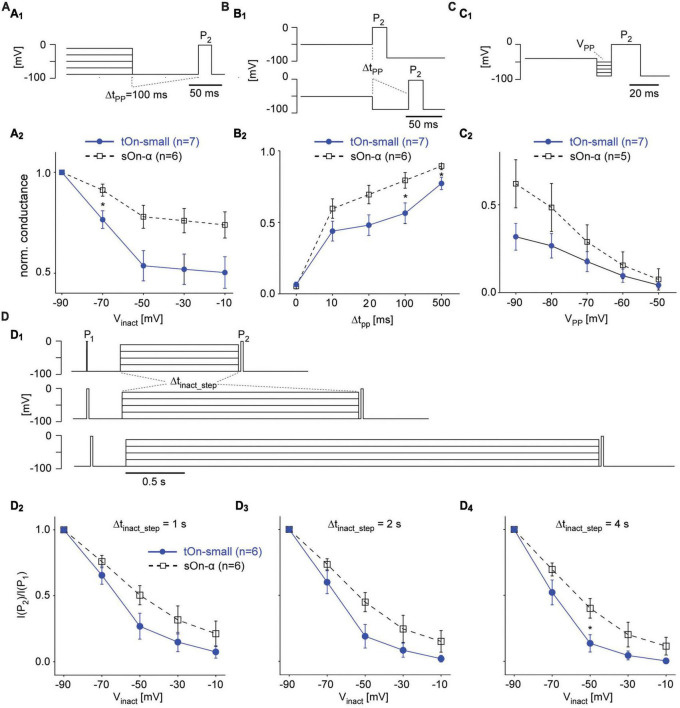
Steady-state slow inactivation of VGSCs shapes tOn-small RGC responses. **(A)** Same protocol as in Figure 5B, but with a 100-ms hyperpolarizing pre-pulse (V_*PP*_ = –90 mV) inserted between inactivation step and test pulse P_2_; P_1_ not shown **(A_1_)**. Normalized conductance as a function of inactivation step voltage for tOn-small (filled circles) and sOn-α RGCs [open squares; **(A_2_)**]. **(B)** Like in **(A)** but with pre-pulse duration varied (Δt_*pp*_ = 0, 10, 20, 100, and 500 ms) for a single inactivation step voltage (V_*inact*_ = –50 mV). **(C)** Like in **(A)** but with fixed Δt_*pp*_ = 10 ms and V_*inact*_ = –40 mV, while varying pre-pulse voltage (V_*PP*_ = –90 to –50 mV). **(D)** Same protocol as in Figure 5B but with the duration of the inactivation step (Δt_*inact_step*_) varied **(D_1_)**. Test pulse current ratio (I(P_2_)/I(P_1_)) as a function of V_*inact*_ for Δt_*inact_step*_ = 1 **(D_2_)**, 2 **(D_3_)**, and 4 s **(D_4_)**. Wilcoxon rank sum test was used for determining statistical significance (* = *p* < 0.05) between different conditions in **(A–D)**.

Our modelling predicts that the Na^+^ channel desensitization observed in tOn-small cells may result from differences in VGSC activation, inactivation and/or recovery from inactivation (Carter and Bean, 2011). To test this experimentally, we recorded Na^+^ currents in these cells and, for comparison, in sOn-α cells (Figures 5, 6). To isolate VGSCs, we blocked voltage-gated K^+^ channels with Cs^+^ and TEA in the electrode solution and voltage-gated Ca^2+^ channels with Cd^2+^ in the extracellular solution (see section “2 Materials and methods”). With voltage-step protocols to characterize VGSC activation (Figure 5A1) and inactivation (Figure 5A2) we found VGSCs in tOn-small cells to activate at higher potentials than those in sOn-α cells (Figure 5A3; tOn-small, −51.1 ± 3.9 mV, *n* = 6; sOn-α, −67.5 ± 3.2 mV, *n* = 7; at 5% activation estimated from sigmoidal fit), providing a likely explanation why a substantial depolarisation (approx. >15 mV from *V*_*Rest*_) is required to trigger spikes in tOn-small cells. This higher activation threshold is also consistent with the tOn-small cells’ selectivity for high contrasts. However, the inactivation profiles were almost identical (Figure 5A3) for the used step protocol, suggesting that the VGSCs in the two cell types share similar steady-state inactivation properties.

### 3.7 Voltage-gated Na^+^ channels in tOn-small RGCs need stronger hyperpolarization and longer times to recover from inactivation

It is known that VGSCs can undergo inactivation at different timescales and that their recovery from different states of inactivation may differ in its time- and voltage-dependence (Tsai et al., 2011; Martiszus et al., 2021). Hence, we next wanted to look at these aspects in the two cell types. To this end, we probed the cells with two test pulses (P_1_, P_2_), with P_2_ following a 1 s “inactivating” voltage-step and a short 20 ms hyperpolarizing pre-pulse (Figure 5B1). While the previous protocol (Figure 5A2) tested the combined effects of fast and slow inactivation, the insertion of a pre-pulse enables VGSCs to recover from fast inactivation and, hence, allowed us to probe the effect of slow inactivation (Kim and Rieke, 2003; Silva, 2014). In the presence of this pre-pulse, the inactivating step reduced the test pulse response in both RGC types, but much more so in tOn-small cells (Figure 5B2). This suggests that the VGSCs’ slow inactivation differed between the two cell types with respect to voltage-dependence, with the VGSCs in tOn-small displaying consistently stronger inactivation for step voltages ≥−70 mV.

To probe the time-dependence of recovery from inactivation, we first applied a pulse-train protocol with two different frequencies (8 and 20 Hz, Figures 5C1, C2), mimicking the activation by spikes (Kim and Rieke, 2003). We quantified VGSC inactivation by calculating the ratio between the Na^+^ current activated by the i*^th^* pulse (*I*_*Na(i)*_) and that activated by the 1st pulse of a train (*I*_*Na(1)*_). We found that *I*_*Na*_ ratio (*I*_*Na*(*i*)_/*I*_*Na*(1)_) in tOn-small cells dramatically dropped at 20 Hz already for the 2nd pulse to ∼75% and for the 10th pulse to less than 50%, whereas in sOn-α cells, *I*_*Na*_ ratio was only slightly reduced by consecutive pulses (Figure 5C2). This indicates that VGSCs in tOn-small cells may need substantially more time to recover from inactivation compared to those in sOn-α cells.

### 3.8 Steady-state slow inactivation of VGSCs shapes tOn-small RGC responses

To study time- and voltage-dependence of RGC responses in our voltage-clamp experiments more closely, we went back to the two-pulse protocol (Figure 6A1). First, we varied the duration of the hyperpolarizing pre-pulse (Δ*t*_*PP*_) between inactivation step and second test pulse (P_2_) to determine the time-dependence of recovery from slow inactivation (Figure 6B). With Δ*t*_*PP*_0, inactivation was virtually the same for both cell types (*cf.* Figures 5A2, A3), but became significantly different for Δ*t*_*PP*_20*ms* before starting to approach similar values for Δ*t*_*PP*_500*ms* (Figure 6B2), which supports a difference in time-dependence of recovery from slow inactivation. Next, we kept the duration of the pre-pulse constant but varied its voltage (V_*PP*_) to test the voltage-dependence (Figure 6C1). We found tOn-small cells recovered only to approx. half the levels compared to sOn-α cells for all tested V_*PP*_ values (Figure 6C2), arguing for an additional difference in voltage-dependence of recovery. Finally, we changed the duration of the inactivating step (Δ*t*_*inact*_*step*_ of 1, 2, or 4 s; Figure 6D1) to make sure the difference between the cell types were not due to insufficient inactivation of the sOn-α cells; this seemed to have not been the case, as for all tested inactivation durations, tOn-small cells consistently showed stronger inactivation than sOn-α cells (Figures 6D2–D4).

Taken together, our data suggests that the differences observed in the spiking behaviour of tOn-small vs. sOn-α cells at least partially arise from VGSCs in tOn-small cells: These VGSCs (i) activate at a higher threshold and require (ii) stronger hyperpolarization and (iii) more time to recover from steady-state slow inactivation. These results support that idea that properties of the spike generator importantly contribute to the temporal coding properties observed in tOn-small RGCs.

### 3.9 Differences between tON-small and sOn-α cell responses are unlikely due to space-clamp errors

**FIGURE 7:**
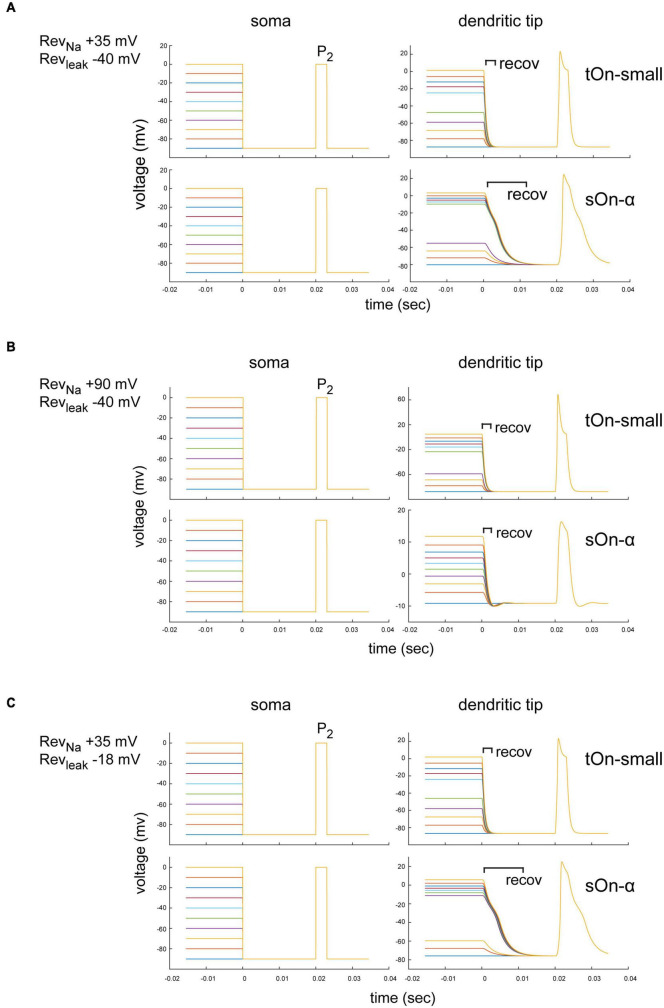
Modelling of space-clamp properties in tON-small and sOn-α retinal ganglion cells. **(A)** Ball-and-stick model showing membrane depolarisations at soma (left) and distal dendritic tips (right) elicited with 3-ms test pulses (P_2,_ –90 to 0 mV) delivered after 1-s inactivating steps (–90 to 0 mV, ΔV = 10 mV, followed by a hyperpolarising step for 20 ms to –90 mV). Membrane potentials elicited by inactivating steps and 3-ms test pulse in a tOn-small cell (top row) and a sOn-α cell (bottom row) are shown. Reversal potentials (Revs) of the “ball-and-stick” model were set to Rev_*Na*_ = +35 mV and Rev_*leak*_ = –40 mV. **(B)** As in **(A)** but model parameters set to Rev_*Na*_ = +90 mV and Rev_*leak*_ = –40 mV. recov, recovery period. **(C)** As in **(A)** but with model parameters set to Rev_*Na*_ = +35 mV and Rev_*leak*_ = –18 mV.

While we isolated the recorded cells from Ca^2+^-dependent synaptic input and blocked voltage-gated K^+^ channels in our voltage-clamp experiments, we cannot exclude that the cells’ morphologies resulted in different space-clamp situations that affected our results. We therefore assessed the effect of morphological differences with computational methods (Poleg-Polsky and Diamond, 2011). We built a ball-and-stick model for both sOn-α and tOn-small cells (see section “2 Materials and methods”; Figure 7) that captured dendritic features, such as diameter and segment length (Fohlmeister and Miller, 1997). We tested the model for different reversal potentials for Na^+^ (Rev_*Na*_) and unspecific leak currents (Rev_*leak*_) and applied a step protocol comprising inactivating voltage steps and the P_2_ test pulse from Figure 5B1. The goal was to test if voltage-clamp errors may have contributed to the differences we observed between the two RGC types in Figures 5B, C.

The specific dendritic morphology may affect the local ion reversal potential because intracellular ion concentrations in high-resistance distal tips are likely to be strongly influenced by ion flux through local channels (vs. diffusion of intracellular solution from the electrode tip at the soma along the dendrite). Therefore, the actual local reversal potential for a particular ion is difficult to estimate but likely different from the theoretical value. We therefore tested for Rev_*Na*_ = +90 mV (the theoretical values defined by our solutions) and Rev_*Na*_ = +35 mV (assuming a higher dendritic Na^+^ concentration). We found that for Rev_*Na*_ = +35 mV, the voltage drop after the depolarization step had a longer time constant in sOn-α vs. tON-small cells, but the voltage recovered to baseline before the P_2_ test pulse (within less than 20 ms) (Figure 7A). Importantly, the modelled voltage amplitudes were very similar for the two RGC types. Therefore, it is unlikely that the smaller current amplitudes observed in tOn-small cells (Figure 5B2) are due to space-clamp issues. For Rev_*Na*_ = +90 mV, the dendritic voltage amplitudes for the P_2_ test pulse were even higher in tOn-small cells than in sOn-α cells (Figure 7B), which are expected to result in higher currents in tOn-small cells, but which was opposite to what we measured experimentally (Figure 5B2).

Similar to Rev_*Na*_, also non-voltage-sensitive leak currents of a neuron can affect the space-clamp properties (Huang et al., 2015). Thus, we modelled the impact of the leak current (Rev_*leak*_) for the condition with the higher dendritic Na^+^ concentration (Rev_*Na*_ = +35 mV) (Figures 7A, C). We estimated Rev_*leak*_ for two extreme ratios of Cl^–^ and Na^+^ conductances: Rev_*leak*_ = −40 mV (Cl^–^:Na^+^ conductance = 12:1) and Rev_*leak*_ = −18 mV (Cl^–^:Na^+^ conductance = 3:1). For both Rev_*leak*_ values, the voltage amplitudes in the dendritic tips were virtually identical (Figures 7A, C), which suggests that the leak currents were unlikely to substantially affect our measurements.

In summary, our modelling results suggest that while space-clamp problems may indeed have affected our Na^+^ current measurements, they also indicate that sOn-α RGCs were expected to be more strongly affected than tOn-small RGCs (i.e., time constant)—which does not match our experimental observations. Therefore, we consider it unlikely that space-clamp issues caused the observed difference in Na^+^ channel inactivation between sOn-α and tOn-small RGCs.

## 4 Discussion

Here we studied the underlying cell-intrinsic mechanisms that encode membrane voltage into spike patterns in two distinct types of RGC in the mouse retina. Recordings of light- and current-elicited voltage responses showed differences in the relationship of membrane voltage and elicited spikes between tOn-small and On-α cells: Unlike in sOn-α RGCs, spiking but not the voltage signal in tOn-small RGCs was very transient and high-contrast selective. Modelling and voltage-clamp recordings revealed that this response pattern of tOn-small cells is largely shaped by stronger desensitisation of the cell’s spike generator, i.e., its VGSCs. Our results support that notion that—in complement with upstream mechanisms and as the last step of the signal retinal processing chain—the intrinsic properties of an RGC can importantly shape the retina’s output to the brain.

### 4.1 Multiple mechanisms can shape RGC response kinetics

Retinal ganglion cell response properties can be shaped at different stages of retinal signal processing. First, RGCs can directly inherit diverse temporal response properties from their excitatory presynaptic partners, the BCs. It has been shown that transient BCs, such as types 5t, 5o, 5i, and XBC, mostly stratify in the central bulk of the IPL (Baden et al., 2013), therefore RGCs may become transient simply by having their dendrites tap into the respective IPL layers to collect transient excitatory input. Interestingly, the tOn-small cells likely correspond to the “6 sn” cells classified in an EM dataset (Bae et al., 2018), which stratify slightly more toward the centre of the IPL than the sOn-α cells, and thus can form synapses with more transient BC types. This may contribute to transient responses in tOn-small RGCs and sustained responses in sOn-α cells. Second, inhibitory AC circuits can sharpen the time course of RGC responses either by shaping the signals at BC axon terminals or RGC dendrites [reviewed in (Zhang and McCall, 2012; Diamond, 2017)]. Third, intrinsic RGC properties, such as kinetics of postsynaptic glutamate receptors, dendritic morphology and active dendritic channels, define the computations executed of distinct RGC types (Brombas et al., 2022). Forth, RGC responses can be shaped by properties of the intrinsic spike generator. For example, in suppressed-by-contrast RGCs, a depolarization block, resulting from a low VGSC conductance, short axonal initial segment (AIS), and selective expression of Na_*v*_ isoforms (see below), defines the cells’ response (Wienbar and Schwartz, 2022). Another example would be direct (neuro)modulation of Na_*v*_ function by reactive oxygen species (ROS), as it was recently shown for sustained RGCs (Smith et al., 2023). In complement, our study suggests that the functional properties of VGSCs expressed in tOn-small cells are one of the key mechanisms that shape these cells’ responses. Further experiments are needed to reveal how synaptic and intrinsic features interact with each other to shape RGC output (Li et al., 2022). In particular, it would be interesting to unravel how excitatory and inhibitory synaptic input sets the operational range of VGSCs in RGCs (Davis and Linn, 2003) and how, in turn, VGSC activity—particularly in the dendrites—modulates integration of neurotransmitter input (Yu, 2006; George et al., 2012; Ran et al., 2020; Brombas et al., 2022).

### 4.2 Spike generator desensitisation and functional consequences

Our results suggest that desensitisation of the spike generator, likely based on slow inactivation of the VGSCs themselves, shapes spike coding in tOn-small cells in two ways: their responses are more transient and their sensitivity for low contrast stimuli is decreased compared with sOn-α cells. In previous studies, it was shown that slow inactivation of VGSCs participates in the adaptation of RGCs to temporal contrasts (Kim and Rieke, 2003; Weick and Demb, 2011). Another study investigating contrast adaptation in RGCs revealed two types of “plasticity” mechanisms: adaptation and sensitisation (Kastner and Baccus, 2011). Interestingly, the plasticity of the adapting type supports encoding of strong stimuli (i.e., high-contrast), whereas plasticity of the sensitising type promotes encoding of weak stimuli (i.e., low-contrast)—very similar to what we found in tOn-small (high contrast-selective) and sOn-α cells (also sensitive to low-contrast stimuli). Therefore, it is likely that desensitisation of the spike generator plays a functionally crucial role in at least three different aspects of RGC signalling: temporal kinetics (transient vs. sustained), contrast adaptation, and limiting the dynamic range for contrast encoding.

In our model, we simulated spike generator desensitization by changing the voltage dependence of the gating functions in a history-dependent manner (see section “2 Materials and methods”). This can be thought as equivalent to slowing the recovery of the VGSCs from inactivation. Also, our voltage-clamp data points at the VGSCs to be involved in defining the response properties of tOn-small cells: compared to sOn-α cells, the Na^+^ currents in tOn-small cells exhibited a higher threshold and recovered more slowly from inactivation. Therefore, that tOn-small and sOn-α cells likely differ in their VGSC complement.

Using RT-PCR, four VGSC α-subunit isoforms have been identified in rodent RGCs: Na_*v*_1.1, 1.2, 1.3, and 1.6 (Fjell et al., 1997). Among other things, these isoforms differ in their persistent current [reviewed in (Goldin, 1999)]: For example, for more positive membrane potentials, the persistent current of Na_*v*_1.6 increases, whereas that of Na_*v*_1.1 decreases. A persistent current that increases with depolarization “pulls” the membrane potential toward the spiking threshold and, thus, may favour sustained spiking. Indeed, a recent study provided evidence that TTX-sensitive Na_*v*_1.6 channels are dominantly expressed in mouse sustained OFF alpha RGCs, whereas suppressed-by-contrast RGCs likely express a different isoform (Wienbar and Schwartz, 2022). Thus, the expression ratio of VGSC isoforms affects the time course of an RGC’s spiking response. In addition, VGSCs are strongly modulated by accessory ß-subunits (reviewed in Goldin, 1999), which can, for example, slow or accelerate inactivation, and shift voltage-dependence. Therefore, not only VGSC properties but also differential ß-subunit expression may also contribute to the differences we observed between tOn-small and sOn-α cells. Like many other proteins in neurons, the intracellular domains of VGSCs are a substrate for phosphorylation, and thus, VGSCs can be functionally modulated. Depending on the phosphorylation/dephosphorylation state, amplitude and inactivation of currents through VGSCs can significantly vary (Numann et al., 1991; Li et al., 1992; Scheuer and Catterall, 2006). In complement, “extracellular” factors such as temperature and concentration of divalent cations in the extracellular space can modulate the voltage dependence of VGSCs (Hahin and Campbell, 1983; Josephson, 1996; Abdelsayed et al., 2015).

In addition to the Na_*v*_1.6 channels, which can be selectively blocked with TTX, it was shown that a small subset of RGCs additionally expresses an unusual TTX-insensitive VGSC α-subunit isoform in the soma and the proximal dendrites: Na_*v*_1.8 (O’Brien et al., 2008). These cells had large somata and were neurofilament-positive, suggesting that alpha (but not tOn-small) cells express Na_*v*_1.8. In contrast to the above VGSC isoforms, Na_*v*_1.8 mediates currents that exhibit very little subthreshold inactivation and recover much faster from inactivation (Cummins and Waxman, 1997; Renganathan et al., 2000). It is possible that channels with such properties contribute to the high contrast sensitivity—for instance, by enabling dendritic spiking (Velte and Masland, 1999)—and the sustained spiking response of sOn-α cells. Nevertheless, we did not observe differences in inactivation between tOn-small and sOn-α cells which argues against a prominent role of Na_*v*_1.8 in sOn-α cells—at least under our experimental conditions. Future pharmacological experiments will unravel how TTX-sensitive and TTX-resistant VGCSs together generate response patterns in individual RGCs.

Finally, VGSCs are precisely arranged in type-specific subdomains along the axon initial segment (Van Wart et al., 2007). These VGSC “bands” appear to vary in length and location (relative to the soma) between RGC types (Fried et al., 2009; Wienbar and Schwartz, 2022). It was suggested that this organization shapes the response properties of the RGC spike generator, including the activation threshold (Fried et al., 2009). Whether the VGSC bands along the axon initial segment of tOn-small and sON-α cells differ remains to be investigated.

### 4.3 Diverse retinal computations are performed by intrinsic properties of RGCs

A great deal of neural computations taking place in the retina ultimately segregates features of the visual stimulus into diverse parallel channels to the brain (reviewed in Wassle, 2004; Gollisch and Meister, 2010; Baden et al., 2020; Kerschensteiner, 2022). Complex interactions involving different sets of interneurons in the two plexiform layers of the retina contribute to this parallel feature extraction (reviewed in Diamond, 2017; Franke and Baden, 2017). Our study highlights that a mechanism at the last step of retinal processing—intrinsic properties of the RGCs—can strongly participate in forming their responses. This is in line with several previous studies showing that spike generation in RGCs is rather diverse in its location and dynamics (see also above): For example, it was reported that Na^+^-based spikes can originate from both the dendrites and the soma region of RGCs (Velte and Masland, 1999; Oesch et al., 2005) and dendritic spikes play an important role in sharpening the directional tuning of DS RGCs (Velte and Masland, 1999; Oesch and Taylor, 2010; Schachter et al., 2010). Moreover, RGCs display very different levels of spike frequency adaptation in response to current injection (O’Brien et al., 2002). Depending on the somatic or dendritic location of the Na_*v*_, the integration properties of RGCs vary, and the cell’s sensitivity can be optimized and tuned for the preferred visual stimulus (Brombas et al., 2022). These studies, together with the present one, support the notion that RGCs are not the passive integrators of upstream signals—as they are still depicted in textbooks—but play an active role in retinal processing.

Our voltage-clamp experiments suggest that the slow VGSC desensitisation in tOn-small RGCs is one of the mechanisms shaping the cells’ distinct light responses. We cannot answer the question, though, where the relevant VGSCs are located: at the soma or along the dendrites. Yet, the subcellular VGSCs distribution pattern shapes how a cell processes synaptic input (Ran et al., 2020; Brombas et al., 2022). The relationship between VGSC channel distribution and kinetics for the overall cell’s light responses needs to be addressed in future experiments. In addition to VGSCs, calcium-gated potassium channels are thought to cause hyperpolarized potentials after depolarization (Lancaster and Adams, 1986; Sah and McLachlan, 1991), and may therefore also contribute to the differential desensitization observed in the two RGC types. Future work is needed to systematically investigate this possibility.

Still, the question arises what the advantage of feature extraction at the very last level of retinal processing may be. Considering the “feature” that is extracted by the tOn-small RGC—selective high contrast signals—one may speculate that this RGC type exclusively relays very strong and reliable events to higher visual areas. Extracting this feature late in the retinal network may bear the advantage that signal-to-noise is at its maximum at the RGC level, providing a suitable high-quality signal to cut off low-contrast responses. Additionally, a feature extraction mechanism intrinsic to RGCs would be largely independent from the highly dynamic inner retinal circuits and could provide “stability” to the output signal. In light of evidence suggesting that the retinal code changes over the full range of illumination conditions (Tikidji-Hamburyan et al., 2014), a spike generator with properties like the one in tOn-small RGCs may provide a “stable” element for encoding high contrast signals across different visual environments.

## Data availability statement

The raw data supporting the conclusions of this article will be made available by the authors, without undue reservation.

## Ethics statement

The animal study was approved by the Institutional Animal Welfare Committee of the University of Tübingen and the MPI for Brain Research, Frankfurt/M. The study was conducted in accordance with the local legislation and institutional requirements.

## Author contributions

LC: Conceptualization, Data curation, Formal analysis, Methodology, Visualization, Writing – original draft. YR: Data curation, Formal analysis, Writing – review and editing. MY: Data curation, Formal analysis, Visualization, Writing – review and editing. OA: Conceptualization, Data curation, Visualization, Writing – review and editing, Formal analysis, Investigation. EB: Data curation, Formal analysis, Writing – review and editing. LH: Data curation, Formal analysis, Writing – review and editing. SH: Conceptualization, Data curation, Formal analysis, Funding acquisition, Project administration, Supervision, Visualization, Writing – original draft. TE: Conceptualization, Formal analysis, Funding acquisition, Project administration, Supervision, Validation, Visualization, Writing – original draft. TS: Conceptualization, Data curation, Visualization, Writing – original draft.

## Footnotes

1
http://imagej.nih.gov/ij/

2
http://museum.eyewire.org

3
https://neuron.yale.edu/neuron/
