# Supplementary material for: Spike desensitisation as a mechanism for high-contrast selectivity in retinal ganglion cells
Source: Front Cell Neurosci. 2024 Jan 10;17:1337768. doi: 10.3389/fncel.2023.1337768 (PMC10806099; doi:10.3389/fncel.2023.1337768)
Supplement: Supplementary file 1 [file Data_Sheet_1.docx]

SUPPLEMENTAL MATERIAL

**Spike desensitisation as a mechanism for high-contrast selectivity**

**in retinal ganglion cells**

Le Chang^1-3^, Yanli Ran^1,2,4^, Mingpo Yang^3^, Olivia Auferkorte^5^, Elisabeth Butz^5^, Laura Hüser^5^, Silke Haverkamp^5,6^, Thomas Euler^1,2^*, and Timm Schubert^1,2^*

*Corresponding authors:

Thomas Euler (thomas.euler@cin.uni-tuebingen.de)

Timm Schubert (timm.schubert@cin.uni-tuebingen.de

Supplementary Figures 1-4


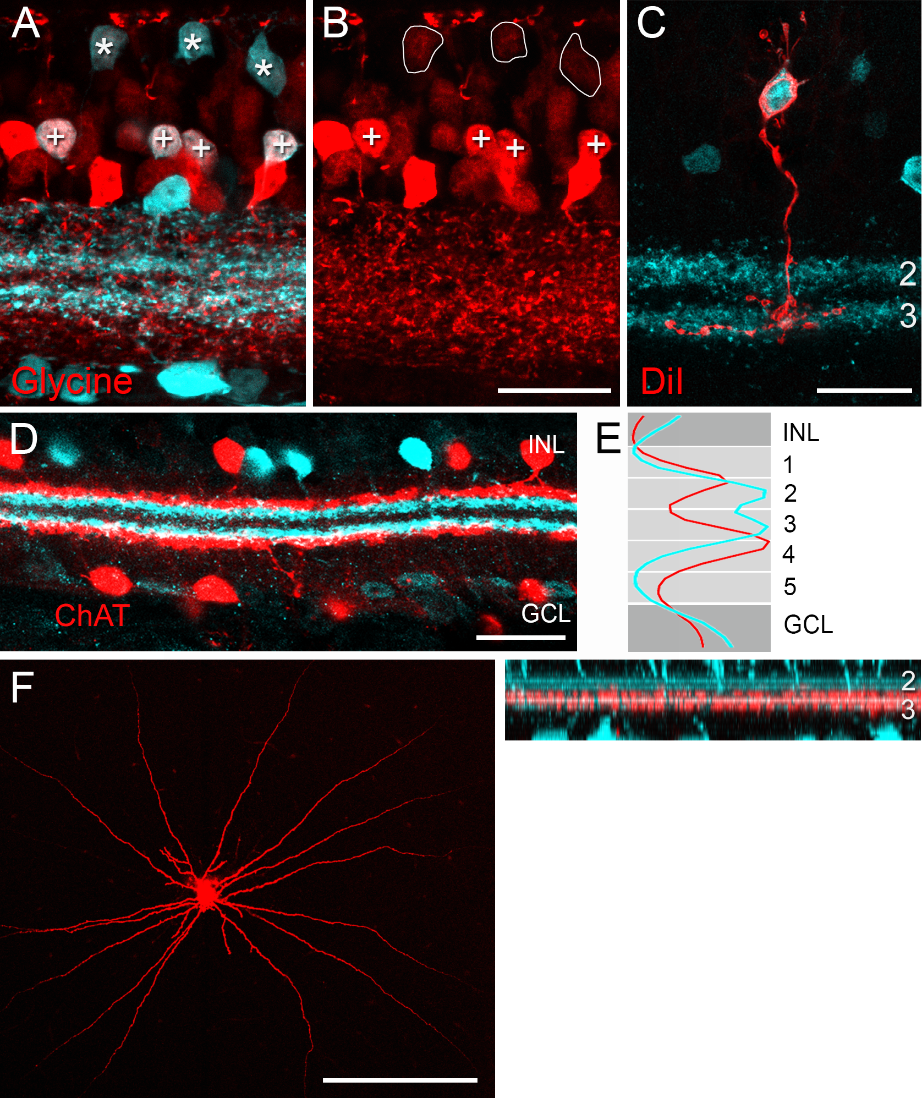


**Supplemental Figure 1** | **EGFP-labelled bipolar and amacrine cells in the Igfbp5 transgenic mouse line** **(related to Fig. 1).** **(A,B)** The INL contained many EGFP-labelled cells, including some GABA-positive ACs (data not shown) and many glycine-positive cells. The latter were comprised of ACs and On cone BCs. Vertical section of Igfbp5 retina double-labelled for EGFP (cyan) and glycine (red). EGFP-labelled ACs in the second-inner row of the INL (+) and ON BCs (*) contain glycine. EGFP-labelled processes extend along sublaminae 2 and 3 of the inner plexiform layer (IPL). **(C)** DiI-injections (n=12 cells) revealed that the EGFP-labelled BCs morphologically corresponded to On cone BC type 5. Example of an individual DiI-injected EGFP-expressing BC with typical type 5 morphology, with axon terminals stratifying in sublamina 3. **(D)** Vertical section double-labelled for EGFP (cyan) and ChAT (red). **(E)** Fluorescence intensity profile along z-axis of a confocal stack of whole mount retina double-labelled for EGFP (cyan) and ChAT (red). **(F)** Analogous dye injections showed that the EGFP ACs included various monostratified, medium- and wide-field cells with On or Off stratification (data not shown). Example of Igfbp5-positive amacrine cell, following DiI filling in a retinal wholemount (left) with dendrites (red) stratifying in sublamina 3 (right). Scale bars: B-D, 20 µm; F, 200 µm.

**

**

**Supplemental Figure 2** | **The mosaic of Igfbp5-positive On amacrine cells (related to Fig. 1). (A)** Reconstruction of a single, representative Igfbp5-positive amacrine cell, following DiI filling in a retinal wholemount. **(B)** Mosaic of 5 neighbouring Igfbp5-positive amacrine cells filled with DiI. **(C)** Illustration of the Igfbp5 plexus serving the retinal patch delimited by the square. The patch contained 27 Igfbp5-positive amacrine cells (black square), to which 5 different representative morphological reconstructions were assigned randomly. Note that although very dense, the plexus is not complete, as dendrites from cell bodies located outside the depicted patch would cross this retinal area. Scale bars: A-C, 200 µm.


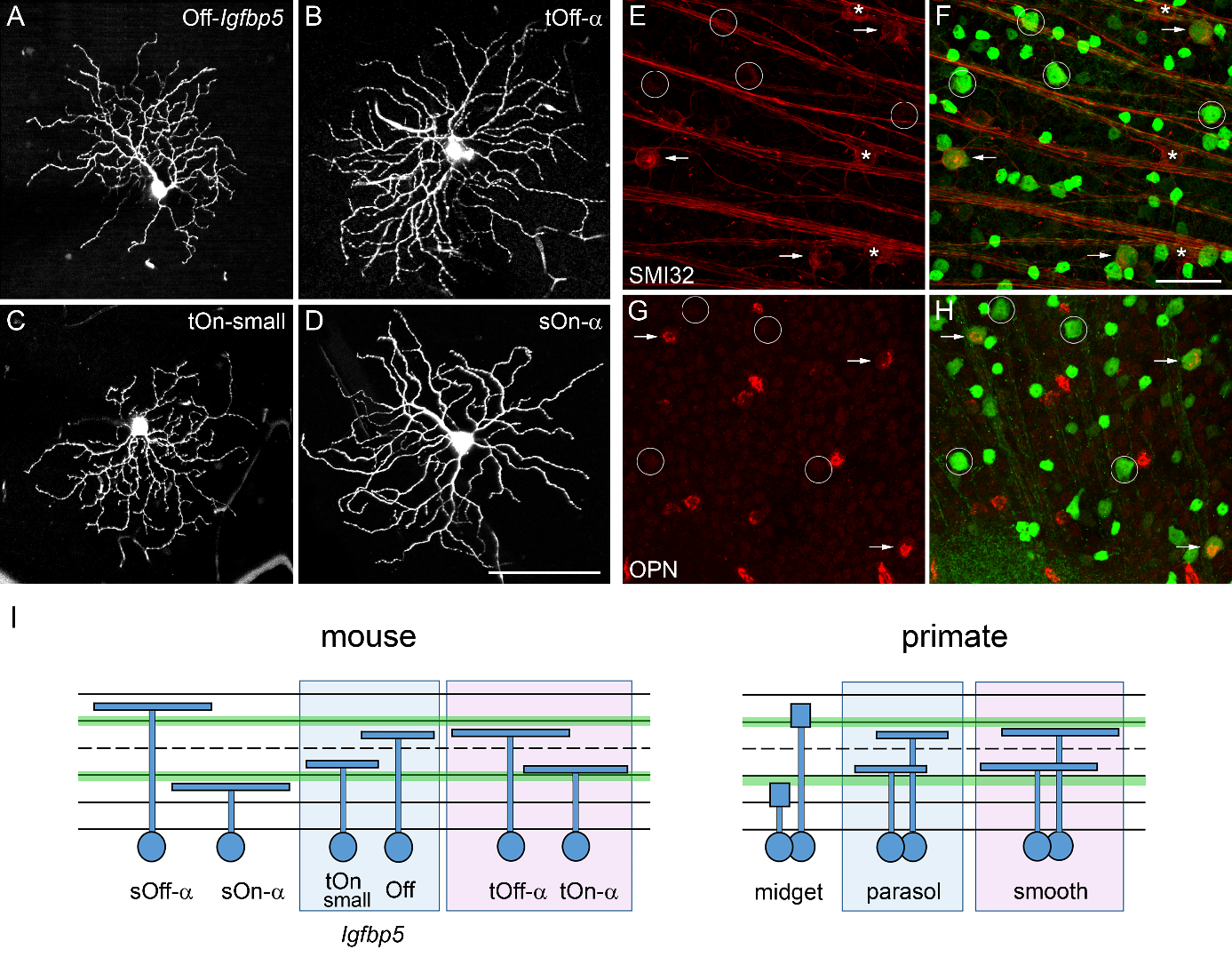


**Supplemental Figure 3** | **Retinal ganglion cells in the Igfbp5 retina (related to Fig. 2). (A-C)** Examples of EGFP-positive RGCs: Off RGC (A), transient Off-alpha (tOff-α) RGC (B), and transient On small (tOn-small) RGC (C). **(D)** For comparison, example of a (Igfbp5-negative) sustained On alpha (sOn-α) RGC. The cells were all collected (and filled with Neurobiotin) from the ventral retina (~0.8 mm from the optic disc). **(E-H)** On Igfbp5-positive RGCs are not immunoreactive for SMI32 and OPN. Igfbp5 retina double-labelled with anti-neurofilament marker SMI32 (red) (Coombs et al., 2006; Bleckert et al., 2014) and anti-GFP (cyan; E,F). sOn-α (asterisks) and tOff-α RGCs (white arrows) were SMI32-positive, whereas tOn-small RGCs (circles) were SMI32-negative. Igfbp5 retina double labelled with anti-osteopontin (OPN, red) and anti-GFP (cyan; G,H). tOff-α RGCs are OPN-positive (white arrows), whereas tOn-small RGCs are OPN-negative (circles). **I**, Possible RGC homologues in mouse and primate retina: Dendritic stratification depth of mouse RGCs within the IPL (left): sOff- α and sOn-α RGCs, Off and tOn-small Igfbp5-positive RGCs, tOff-α and tOn-α RGCs. For comparison, dendritic stratification depth of primate RGCs (right): Off and On midget RGCs, Off and On parasol RGCs, and Off and On smooth RGCs (adapted from Crook et al., 2008). Scale bars: A-D, 100 µm; E-H, 50 µm.


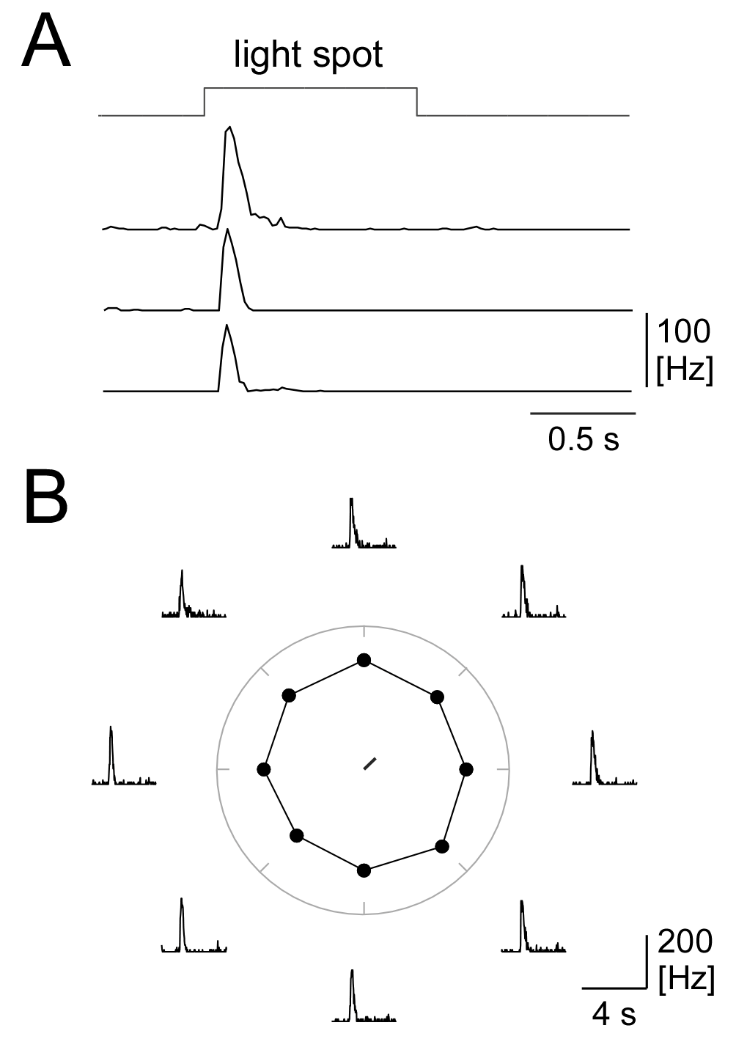


**Supplemental Figure 4 | tOn-small ganglion cells are transient but not direction-selective** **(related to Fig. 3). (A)** Spike responses of three tOn-small RGCs to 1-s step light flash (diameter = 200 µm; average from 10 trials each, 20-ms time bins). **(B)** Spike responses of an exemplary tOn-small cell to bar stimuli moving in 8 directions; responses shown as peri-stimulus time histogram (averages from 10 trials per direction, 20-ms time bins). Centre: Polar plot showing directional tuning curve and vector sum (calculated from the mean spike rate during stimulation for 8 directions) for an exemplary tOn-small RGC.
